# Simplified Diagnosis of Urosepsis by Emergency Ultrasound Combined with Clinical Scores and Biomarkers

**DOI:** 10.2478/jccm-2024-0006

**Published:** 2024-01-30

**Authors:** Alice Nicoleta Dragoescu, Petru Octavian Dragoescu, Andreea Doriana Stanculescu, Vlad Padureanu, Dalia Dop, Mihai Alexandru Radu, Mirela Marinela Florescu, Daniela Teodora Maria, Dan Nicolae Florescu, George Mitroi

**Affiliations:** Faculty of Medicine, University of Medicine and Pharmacy of Craiova, Craiova, Romania; Faculty of Pharmacy, University of Medicine and Pharmacy of Craiova, Craiova, Romania

**Keywords:** urosepsis, ultrasound, neutrophil-to-lymphocyte ratio, procalcitonin, NEWS, SOFA

## Abstract

**Background:**

Urosepsis is a life-threatening medical condition due to a systemic infection that originates in the urinary tract. Early diagnosis and treatment of urosepsis are critical to reducing mortality rates and preventing complications. Our study was aimed at identifying a fast and reliable method for early urosepsis diagnosis and severity assessment by combining prognostic scores such as SOFA and NEWS with ultrasound examination and serum markers PCT and NLR.

**Methods:**

We performed a single-center prospective observational study in the Craiova Clinical Emergency Hospital. It initially analysed 204 patients admitted for sepsis of various origins in our hospital between June and October 2023. Those with urological conditions that were suspected to have urosepsis have been selected for the study so that finally 76 patients were included as follows: the severe cases with persistent hypotension requiring vasopressor were enrolled in the septic shock group (15 patients - 19.7%), while the rest were included in the sepsis group (61 patients - 80.3%). Mortality rate in our study was 10.5% (8/76 deaths due to sepsis).

**Results:**

Both prognostic scores SOFA and NEWS were significantly elevated in the septic shock group, as were the sepsis markers PCT and NLR. We identified a strong significant positive correlation between the NEWS and SOFA scores (r = 0.793) as well as PCT and NLR (r=0.417). Ultrasound emergency evaluation proved to be similar to CT scan in the diagnosis of urosepsis (RR = 0.944, p=0.264). ROC analysis showed similar diagnostic performance for both scores (AUC = 0.874 for SOFA and 0.791 for NEWS), PCT and NLR (AUC = 0.743 and 0.717).

**Conclusion:**

Our results indicate that an accurate and fast diagnosis of urosepsis and its severity may be accomplished by combining the use of simpler tools like emergency ultrasound, the NEWS score and NLR which provide a similar diagnosis performance as other more complex evaluations.

## Introduction

Urosepsis is a life-threatening medical condition characterised by a systemic infection that originates in the urinary tract. Urosepsis can progress rapidly, leading to organ dysfunction, hypotension, and septic shock, making it a medical emergency that requires immediate therapeutic intervention [1]. Prompt diagnosis through clinical evaluation, blood cultures, and imaging is crucial [2]. Serum markers are valuable tools in identifying sepsis at an early stage, allowing for timely intervention. Biomarkers like procalcitonin (PCT), C-reactive protein (CRP), and lactate play pivotal roles in sepsis diagnosis and monitoring. Combining these markers with clinical assessment enhances the accuracy of sepsis diagnosis and guides timely treatment decisions [3,4,5]. The Neutrophil-to-Lymphocyte Ratio (NLR) is another valuable assessment in sepsis, reflecting the balance between the inflammatory response and immune suppression. Elevated NLR is associated with increased severity and mortality in septic patients [6,7].

As the current 2021 International Guidelines for Management of Sepsis and Septic Shock recommend against using the qSOFA score (quick Sequential Organ Failure Assessment) compared with the SIRS (Systemic Inflammatory response Syndrome), NEWS (National Early Warning Score) or MEWS (Modified Early Warning Score) scores as a single screening tool for sepsis or septic shock, we decided to use the NEWS score as screening tool for sepsis and septic shock diagnostic [8,9]. NEWS is a composite score including seven vital parameters (respiratory rate (RR), heart rate (HR), systolic blood pressure (SBP), pulse oximetry (SpO_2_), temperature (T°), and the Alert-Verbal-Pain-Unresponsive (AVPU) scale) utilised to quantify a patient’s condition. A total score of ≥ 5 appears to be well suited to identify deterioration in acutely ill patients. It is one of the most commonly used early warning scores (EWS) worldwide [9,10,11,12,13].

The more complex Sequential Organ Failure Assessment (SOFA) score is a vital tool in the diagnosis and assessment of sepsis and its associated organ dysfunction in the intensive care units. Developed to monitor the progression of organ failure in critically ill patients, the SOFA score assigns points based on the dysfunction of six organ systems: respiratory, cardiovascular, hepatic, coagulation, renal, and neurological. Additionally, the SOFA score is a component of the Sepsis-3 criteria, which provides a more refined and accurate definition of sepsis, emphasising organ dysfunction as a key diagnostic element [14].

Ultrasound imaging plays a crucial role in the diagnosis and management of urosepsis. It allows clinicians to visualise the urinary tract and identify potential sources of infection, such as kidney stones or obstructed urine flow which can contribute to urosepsis development. Moreover, ultrasound is non-invasive, readily available, and lacks ionizing radiation, making it a safe and efficient diagnostic tool [15]. Abdominal computed tomography (CT) imaging is of paramount importance in the diagnosis and management of sepsis, as it offers critical insights into the source, extent and complications of abdominal infections. Timely and accurate identification of the infective focus is essential for initiating appropriate treatment strategies, which can significantly impact patient outcomes [16, 17].

Our study aims at identifying an easy, fast and reliable method for early urosepsis diagnosis and severity assessment by combining prognostic scores such as SOFA and NEWS with ultrasound or CT examination and various serum markers like PCT, CRP or NLR.

## Material and Method

This was a single-centre prospective observational study performed in the Craiova Clinical Emergency Hospital. Ethics committee approval no. 79/07.04.2023 was obtained prior to study initiation. We initially analysed 204 patients admitted for sepsis of various origins in our hospital between June and October 2023. We diagnosed urinary sepsis and septic shock according to Sepsis-3 criteria as follows: sepsis – suspected or documented urinary tract infection and SOFA score ≥ 2; septic shock – sepsis plus vasopressor therapy needed to maintain MAP ≥ 65 mmHg plus lactate ≥ 2 mmol/L (18 mg/L) despite adequate fluid resuscitation [14]. Patients were subsequently assessed by abdominal ultrasound and abdominal CT scan. Those with urological conditions that were suspected to have urosepsis have been selected for the study. We included in our study 76 patients admitted for urinary sepsis. Including criteria for the study were: age ≥ 18, sepsis diagnosed by the NEWS and/or SOFA scores as well as a urinary tract condition (pyelonephritis, obstructive uropathy, stone disease, pyonephrosis, indwelling urinary catheters, etc.) or a recent endoscopic or percutaneous urological procedure. Excluding criteria were subjects younger than 18 years old, pregnant females, patients with compromised immunity, advanced cancers or other terminal illnesses. Before study enrolment, all patients (or close relatives if the patient was unconscious or unable) were informed about the study and provided signed informed consent. The large majority of patients underwent urological procedures intended to decompress and drain the urinary tract immediately after hospital admission and initial evaluation: ultrasound guided percutaneous nephrostomy or perirenal drainage, ureteral JJ stent placement or replacement, suprapubic or urethro-vesical catheterisation.

Medical history as well as clinical signs and symptoms and vital signs were collected from all patients. Clinical examination and local exam was completed upon hospital admission. Regular venous blood sampling was drawn after admission for standard blood tests: haematology, biochemistry, arterial blood gases, serum lactate, as well as blood inflammation markers: C-reactive protein (CRP), and procalcitonin (PCT). As soon as possible after hospital admission, two pairs of blood cultures (aerobic and anaerobic) as well as urinalysis and urine culture were sampled before starting empiric antibiotic management. We calculated the NLR value that is known to normally range between 1 and 3 [7]. Several studies have shown its predictive power in assessing the risk of sepsis progression and guiding the treatment [19]. The NEWS and SOFA were calculated upon admission for all study patients. C-reactive protein (CRP) values were measured upon hospital admission using immuno-turbidimetry method.

Procalcitonin was analysed on Elecsys Cobas e601 Roche^®^, which is a fully automated analyser that uses the electro-chemiluminescence immunoassay (ECLIA) principle. It is designed for both quantitative and qualitative in vitro assay determinations.

Ultrasound examinations were performed for all patients using a portable ultrasound platform that included the Philips Lumify^®^ (4-1 MHz) S4-1 ultrasound transducer especially designed for FAST ultrasound evaluation in conjunction with a handheld tablet. During ultrasound evaluation of the urinary tract, we assessed the status of the upper urinary tract (hydronephrosis, urinary sones, indwelling internal catheters) as well as the lower urinary tract (bladder stones, diverticula, urinary retention, enlarged prostate). Abdominal and pelvic CT scan was performed for all patients. We identified urinary tract obstructions or renal stones as well as lower urinary tract conditions that are instrumental in finding the source of urosepsis.

All patient data was collected from medical charts, lab results, imaging reports and other source documents and logged into excel spreadsheets. Normality of data samples was assessed by the Kolmogorov-Smirnov test. Normally distributed data was analysed using the Student t-test, while the non-parametric analysis was performed using the Mann-Whitney U test. The Pearson r correlation coefficient was used for clinical correlations evaluation while the ROC curve analysis was employed for diagnostic performance assessment. Significance level was established at .05 for all statistical tests. Statistical analyses were performed using Med-Calc software for Windows, version 22.013 (MedCalc Software, Ostend, Belgium).

## Results

Our 76 patients with urosepsis were divided in two severity groups as follows: the severe cases were enrolled in the septic shock group (15 patients - 19.7%), while the rest were included in the sepsis group (61 patients - 80.3%). Mortality rate in our study was 10.5%.

Patients' characteristics, inflammatory markers, sepsis biomarkers, and other significant biological parameters are summarized in Table 1. Overall average patient age was 63.4 years, but it was significantly higher for patients with septic shock (70.3 vs 61.7 years, p=0.0145). Most of the patients were males (68.4%, 52/76 subjects), but no significant difference was found between the two sexes regarding disease severity. Regarding vital signs, overall mean arterial pressure (MAP) was 80 ± 11, with significantly lower values for patients with septic shock compared to subjects with sepsis (67 vs 83, p < 0.001) as expected. Average heart rate was overall within normal limits (82 ± 18 beats/min), but significantly higher for patients with septic shock (98 vs. 78 beats/min, p=0.0189, while mean respiratory rate was overall elevated (23 breaths/min) and also significantly higher for patients with septic shock (27 vs 22 breaths/min, p=0.027). Glasgow coma score (GCS) was lower for the patients with septic shock (11.2 vs 12.7, p=0.005).

**Table 1.** Patient demographic and biological parameters and scores with comparison between those with sepsis and septic shock.

**Parameter**	**Total (n=76)**	**Urosepsis (n= 61)**	**Septic Shock (n=15)**	**p=**
Age (years)	63.4 ±15.1	61.7 ±15.7	70.3 ±10.2	0.015*
Sex (M/F)	52/24	43/18	9/6	0.433#
SOFA score	6.1 ± 3.2	5.3 ± 2.3	9.4 ± 2.9	< 0.001*
NEWS	9.8 ± 2.9	9.1 ± 2.3	12.6 ± 3.4	0.0013*
GCS	12.4 ±1.7	12.7 ±1.6	11.2 ±1.7	0.005*
MAP (mmHg)	80 ± 11	83 ± 9	67 ± 6	< 0.001*
HR (beats/min)	82 ± 18	78 ± 13	98 ± 28	0.019*
RR (breaths/min)	23 ± 7	23 ± 7	23 ± 7	0.027*
WBC (×10^3^/mm^3^)	18 [15–22]	17 [14–20]	21 [19–23]	0.412^
NEU (×10^3^/mm^3^)	15 [13–18]	14 [12–17]	18 [15–19]	0.313^
LYM (×10^3^/mm^3^)	1.6 [1.2–2.1]	1.6 [1.3–2.1]	1.5 [1.2–1.8]	0.596^
NLR	10.3 ± 3.2	9.8 ± 3.0	12.2 ± 3.4	0.023*
PLT (×10^3^/mm^3^)	133 ± 49	140 ± 50	108 ± 32	0.042*
C-Reactive Protein (mg/l)	124 [92–145]	124 [94–143]	136 [79–153]	0.697^
Procalcitonin (PCT) (ng/ml)	13.0 ±5.7	11.8 ±4.6	18.0 ±7.4	0.007*
ESR (mm/h)	40.4 ±16.4	38.9 ±16.9	42.3 ±14.9	0.587*
Lactate (mmol/l)	1.8 ± 1.4	1.6 ± 1.2	2.7 ± 1.8	0.039*
Creatinine (mg/dl)	1.9 ± 1.3	1.9 ± 1.4	2.1 ± 1.1	0.619*
Bilirubin (mg/dl)	1.8 ± 1.0	1.7 ± 1.0	2.2 ± 0.9	0.085*

SOFA - Sequential Organ Failure Assessment, NEWS - National Early Warning Score, GCS – Glasgow Coma Scale, MAP – mean arterial pressure, HR – heart rate, RR – respiratory rate, WBC – white blood cells, NEU – neutrophils, LYM – lymphocytes, NLR – neutrophil-to-lymphocyte ratio, PLT – platelet, ESR – erythrocyte sedimentation rate, Data presented as mean and standard deviation, ratio or median and inter-quartile range depending on data type and distribution

(*= Student t-test, # = Chi-square test, ^= Mann-Whitney U test).

Urological conditions or complicating factors associated with urosepsis were identified in 72 patients (94.7%): ureteral obstruction with uni/bilateral hydronephrosis (53 cases - 69.7%), urinary tract stones (48 patients - 63.2%), indwelling urinary catheters: double J ureteral stent (29 patients - 38.2%) or percutaneous nephrostomy catheter (19 patients - 25%) as well as history of recent endourologic surgery (12 patients - 15.8%). Most frequent non-urological associated comorbidities were diabetes mellitus (22.3%) and high blood pressure (31.5%), but no significant correlation was found to urosepsis severity.

Significant differences between average values in subjects with urosepsis and septic shock were identified for platelets (140 vs 108 × 10^3^/mm^3^, p=0.042) and serum lactate (1.6 vs 2.7 mmol/l, p=0.039) only. We further calculated the neutrophil-to-lymphocyte ratio (NLR) and found overall elevated values (10.3 ± 3.2) with significantly higher ratios for patients with septic shock (12.2 vs. 9.8, p=0.023). Regarding microbiological data, urine cultures were positive in 64 patients (84.2%) while blood cultures were positive for 41 patients only (53.9%). Patients with no available positive culture were diagnosed as suspected urosepsis on positive urinalysis results (pyuria, or ≥ 10 WBC/HPF of unspun urine) associated with clinical and/or imagistic suspicion of a urological condition. CRP was higher for patients with septic shock, but the difference was not significant (median value 136 vs. 124 mg/l, p=0.697), while PCT was significantly higher (18.0 vs. 11.8 ng/ml, p=0.007). In clinical practice, the two proteins are widely used for both sepsis diagnosis and severity assessment.

The SOFA score was elevated in all urosepsis patients (as it was an inclusion criterion at ≥ 2) with an average value of 6.1 ± 3.2. The score was significantly higher for patients with septic shock compared to those with urosepsis (9.4 vs 5.3, p < 0.001). NEWS score was similarly raised above normal limit for all study subjects and significantly higher for the more severe patients (12.6 vs. 9.1, p=0.0013) (Table 1).

**Fig. 1.**
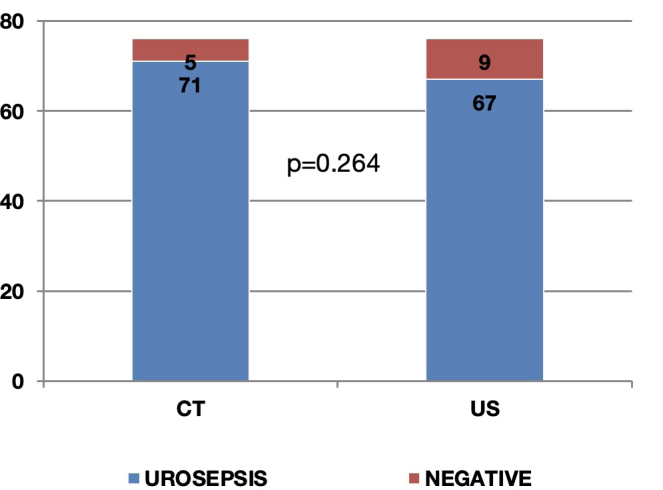
Comparison between computerised tomography (CT) and ultrasound evaluation (US) regarding the number of patients diagnosed with urosepsis shows the non-inferiority of US vs CT (RR=0.944, 95%CI = 0.852–1.045, p=0.264).

We further analysed the value of emergency ultrasound evaluation compared to computerised tomography (CT) in the early diagnosis of urosepsis, as ultrasound is significantly cheaper, easier to perform and faster than CT scan. The number of patients identified with urinary tract abnormalities that may constitute the source for urosepsis were noted for both imaging tests as follows: 71 patients from the total of 76 had at least one abnormality on CT scan (93.4%) while ultrasound emergency examination identified the cause for urosepsis in 67 patients only (88.2%) and no cause in 9 patients (Figure 1). While it may be obvious that CT is superior to ultrasound, we tried to assess whether it is significantly so. We therefore calculated the relative risk (RR) for a patient diagnosed by ultrasound only to have an inaccurate diagnosis versus the patient diagnosed by CT scan. The RR was 0.944 (with 95%CI = 0.852–1.045, p=0.264) and NNT (number needed to treat) of 19. These results confirm the non-inferiority of ultrasound vs. CT scan in the diagnosis of urosepsis and indicate that only one in 19 patients (5%) would indeed benefit from CT scan evaluation following ultrasound.

**Fig. 2.**
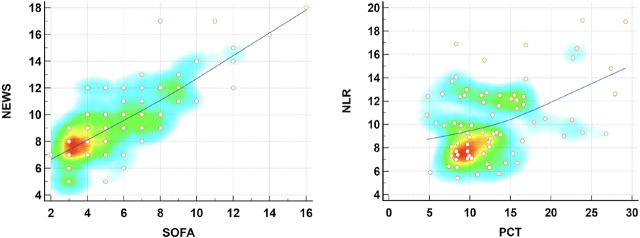
Correlation scatter graphs and heat-maps for the 2 prognostic scores (SOFA - Sequential Organ Failure Assessment and NEWS - National Early Warning Score, r = 0.793, 95%CI = 0.692 – 0.864, p < 0.0001) as well as the two important markers (PCT and NLR, r = 0.417, 95%CI = 0.211 – 0.587, p=0.0002) show strong and significant correlations.

Subsequently we analysed the potential statistical correlations between the most important markers and scores in our study by evaluating the Pearson correlation coefficient (r) with its 95%CI and statistical significance (Figure 2). The most significant positive correlation was identified between the NEWS and SOFA scores with r = 0.793 (95%CI = 0.692 – 0.864, p < 0.0001) which is expected as both scores evaluate the clinical and biological status of the septic patient and confirms a consistent positive trend in prognostic assessment for both. We may therefore infer that NEWS may be used as a substitute for the SOFA score in certain emergency situations. Another significant positive correlation was identified between the sepsis markers NLR and PCT (r = 0.417, 95%CI = 0.211 – 0.587, p=0.0002).

We further identified a moderate correlation between PCT and the SOFA score (r = 0.334, 95%CI = 0.117 – 0.520, p=0.0032), but not the NEWS score (r = 0.098, 95%CI −0.130 – 0.316, p=0.400). No significant correlations were identified however between NLR and any of the sepsis scores SOFA (r = 0.101, 95%CI −0.127 – 0.319, p=0.385) and NEWS (r = 0.069, 95%CI −0.159 – 0.290, p=0.552).

In order to evaluate the severity diagnostic performance of each marker and score, we performed the ROC (receiver operating characteristic) curve analysis for each parameter followed by a pairwise comparison of all ROC curves. The results confirmed the SOFA score is the most performant in the urosepsis severity assessment with an area under curve (AUC) of 0.874 and 87% Sensitivity (Sn) with 72% Specificity (Sp) for a cut-off value of the score > 6 (p<0.0001), followed by the NEWS score with AUC = 0.791, Sn= 67%, Sp = 74%, cut-off value > 10, p <0.0001. The sepsis markers PCT and NLR also performed well with AUC of 0.743 and 0.717 respectively, while CRP had a non-significant AUC of only 0.543 (Table 2).

**Table 2.** Comparison of AUC values with standard error (SE), 95%CI of AUC, Sensitivity (Sn), Specificity (Sp), cut-off value and statistical significance (p) for all analysed parameters: SOFA - Sequential Organ Failure Assessment, NEWS - National Early Warning Score, PCT – procalcitonin, NLR - neutrophil-to-lymphocyte ratio, CRP – C-reactive protein.

**Variable**	**AUC**	**SE**	**95% CI**	**Sn**	**Sp**	**Cut-off**	**p=**
SOFA	0.874	0.043	0.778 to 0.939	87%	72%	6	<0.0001
NEWS	0.791	0.065	0.682 to 0.876	67%	74%	10	<0.0001
PCT	0.743	0.080	0.629 to 0.836	67%	74%	13.6	*0.0025*
NLR	0.717	0.072	0.603 to 0.815	80%	62%	9.9	*0.0023*
CRP	0.543	0.099	0.425 to 0.658	67%	59%	127	0.664

**Fig. 3.**
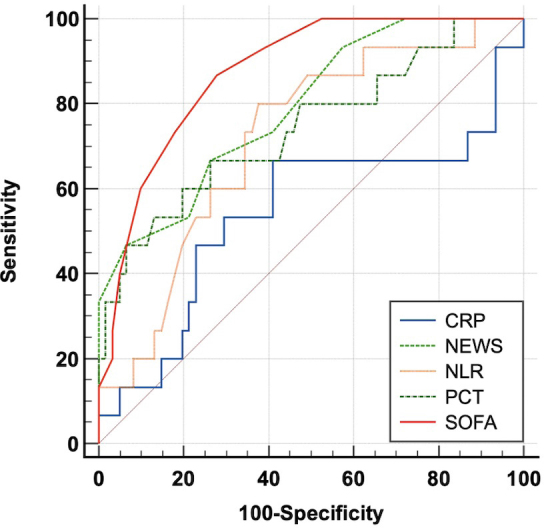
ROC curve comparison for all study parameters: SOFA, NEWS, PCT, NLR, CRP. (SOFA - Sequential Organ Failure Assessment, NEWS - National Early Warning Score, PCT – procalcitonin, NLR - neutrophil-to-lymphocyte ratio, CRP – C-reactive protein).

The pairwise comparison of the ROC curves (Figure 3) showed no statistically significant differences between SOFA, NEWS, PCT and NLR while CRP proved to be inferior to both NEWS and SOFA. However, the AUC values listed above confirm that SOFA followed by NEWS score are slightly better than both PCT and NLR. Moreover, NLR showed a similar diagnostic performance to PCT (AUC 0.717 vs 0.743, p= 0.767).

These results seem to indicate that we can identify useful alternatives to the *classical* way of diagnosing and assessing urosepsis that usually includes complex scores like the SOFA score, expensive markers like PCT and time consuming and complicated imaging tests. We can therefore propose using the significantly simpler, cheaper and faster counterparts like the NEWS score combined with the NLR measurement and emergency ultrasound evaluation as they have proved to be similarly efficient and useful in evaluating these patients.

## Discussions

In urosepsis diagnosis and management, several diagnostic tools have been assessed for their diagnostic performance, including the NEWS and SOFA scores, NLR, procalcitonin and ultrasound imaging. These tools play essential roles in evaluating the severity, prognosis, and early detection of urosepsis. Each of these tools offers unique insights and advantages.

The NEWS score is a clinical tool that has gained widespread recognition for its effectiveness in identifying deteriorating patients, including those at risk of sepsis [9]. Several studies have demonstrated the effectiveness of NEWS in urosepsis diagnosis. A study by Doungsuriya et al. found that NEWS was significantly higher in urosepsis patients compared to non-urosepsis patients, highlighting its potential to identify those at risk [20]. Likewise, the NEWS score was significantly increased in our study for the more severe patients (12.6 vs. 9.1, p=0.0013), while having a very good AUC of 0.791 with a cut-off value above 10.

Other studies such as those by Vincent et al. [21] and Ferreira et al. [22] have indicated the ability of the SOFA score to effectively predict the severity and prognosis of urosepsis. Ferreira et al. has consistently shown that higher SOFA scores upon admission have been correlated with increased mortality rates and longer hospital stays, highlighting its crucial role in risk stratification and therapeutic decision-making [22]. In our study, the SOFA score was elevated in all urosepsis patients with an average value of 6.1. It was significantly higher for patients with septic shock compared to those with urosepsis (9.4 vs 5.3, p < 0.001). Additionally, our study confirmed that the SOFA score is the most performant in the urosepsis severity assessment with an AUC of 0.874 and very good Sensitivity (87%) and Specificity (72%), cut-off value > 6. We further identified a significant strong correlation between the two scores (r=0.793) suggesting a similar prognostic value for both which implies that the NEWS score could be used as a substitute for the SOFA score in certain situations outside the ICU.

NLR is a straightforward and accessible assessment that reflects systemic inflammation and immune response. Studies have consistently shown that an elevated NLR at admission is associated with a more severe clinical course in urosepsis. In a study by Hwang et al. NLR proved good sensitivity and specificity as an indicator of the inflammatory response in urosepsis patients [23]. In a similar way, we found that NLR was overall elevated while significantly higher for patients with septic shock (12.2 vs. 9.8).

Procalcitonin is another biomarker that has gained significant attention in urosepsis diagnosis. Several studies have demonstrated PCT’s high specificity for bacterial infections helping clinicians establish early and accurate diagnoses [4, 24]. Other studies performed by Jiang et al. and Çilesiz et al. have highlighted its diagnostic potential. Elevated procalcitonin levels are specific to bacterial infections, making it valuable in distinguishing urosepsis from other causes of systemic inflammation [25, 26]. Çilesiz et al. have also indicated that procalcitonin levels are significantly elevated in urosepsis patients compared to those with other causes of systemic inflammation [26]. Their results are consistent with our findings that showed significantly increased PCT values for patients with severe forms of urosepsis. However, a growing body of research suggests that NLR may be a non-inferior alternative to PCT. In fact, NLR has gained increasing attention for its diagnostic accuracy in urosepsis, potentially offering a more cost-effective and widely available option. A recent study conducted by Verma et al. demonstrated that NLR had similar efficiency to procalcitonin in identifying urosepsis, suggesting it can be a reliable alternative for early diagnosis [27]. Another study by van Nieuwkoop et al. found that while procalcitonin was the most specific biomarker in urosepsis, NLR exhibited a comparable diagnostic performance, making it a valuable tool for urosepsis diagnosis [28]. Similarly, our ROC analysis showed a similar diagnosis performance for NLR (AUC = 0.717) and PCT (AUC = 0.767) with no significant difference between them, but with a good, significant correlation (r=0.417) that allows us to suggest they may be used as substitutes for each other with NLR obviously being the easier, faster and cost-effective alternative.

Regarding the value of ultrasound imaging in urosepsis, studies like that by Angeletti et al. have underlined its significance in clinical decision-making. Its non-invasive nature and effectiveness in identifying urinary tract abnormalities make it a valuable tool for timely and informed decision-making in urosepsis patient management [29]. Our analysis concluded that ultrasound is indeed a valuable diagnostic tool in urosepsis and is similar to the more complex and expensive CT evaluation. We actually found that only one in 19 patients (5%) would benefit from CT scan evaluation following ultrasound so that it would much simpler, faster and cost-effective to limit the initial emergency evaluation of these patients to ultrasound only.

The strength of our study is that it has direct clinical relevance, as our results suggest a simple, useful and clinically feasible as well as cheaper alternative to the diagnostic methodology we currently use for urosepsis with direct implications in patient outcome improvement. However, there are certain limitations that we identified for our study that are represented by the rather small patient sample, the lack of repeated measurements of blood tests and sepsis markers as well as repeated imaging tests. These are all due to the limited amount of time and funding available at the time of study initiation. An ideal study would obviously include more patients over a longer inclusion period and would probably require more markers and tests, but that would also complicate the study analysis further. Moreover, the lack of homogeneity of urosepsis itself that is due to the various complicating urinary tract conditions may constitute a significant shortfall for this kind of study so that maybe more strict enrolment conditions should be applied in order to obtain more accurate results.

We conclude that an accurate and fast diagnosis of urosepsis and its severity may be accomplished by combining the use of simpler tools like NLR as a sepsis marker, the NEWS score for clinical assessment and emergency ultrasound examination, which together provide a similar diagnosis performance as their more complex and expensive counterparts.
